# Utility of venoactive compounds in post-thrombotic syndrome: A systematic review

**DOI:** 10.1016/j.jvsv.2025.102228

**Published:** 2025-03-16

**Authors:** Monika L. Gloviczki, Julianne Stoughton, Alessandra Puggioni, Peter Gloviczki, Joseph D. Raffetto

**Affiliations:** a Department of Internal Medicine and Gonda Vascular Center, Mayo Clinic, Rochester, MN; b VASA LLC, Scottsdale, AZ; c Division or Vascular and Endovascular Surgery, Massachusetts General Hospital, Boston, MA; d East Vein and Lymphatic Center, New York, NY; e Division of Vascular and Endovascular Surgery, Mayo Clinic, Rochester, MN; f Heart and Vascular Center, Semmelweis University, Budapest, Hungary; g Section of Vascular Surgery, VA Boston Healthcare System, Brigham and Women’s Hospital, Boston, MA

**Keywords:** Chronic venous disease, Chronic venous insufficiency, Deep vein thrombosis, Diosmin, Hidrosmine, Micronized purified flavonoid fraction, Post-thrombotic syndrome, Rutosides, Sulodexide, Systematic review, Venoactive compounds, Venous leg ulcer

## Abstract

**Background:**

Post-thrombotic syndrome (PTS) has been highly prevalent; over 50% of the patients develop PTS after lower extremity acute deep vein thrombosis. Venoactive compounds (VACs) have been recommended for decades for patients with chronic venous insufficiency, including PTS. The objective of our study was to perform a systematic review to determine the quality of evidence on the utility of VACs for both prevention and treatment of PTS.

**Methods:**

A systematic review was conducted to search the literature between January 1, 1980, and July 14, 2023, for venoactive drugs or medications, deep vein thrombosis, and PTS using PubMed, MEDLINE, life science journals, and the Cochrane Library. Only randomized controlled trials (RCTs) published in English were included in this review. The Preferred Reporting Items for Systematic Reviews and Meta-Analyses (PRISMA) guidelines and the revised Cochrane risk-of-bias tool for RCTs were used.

**Results:**

Ninety-four references were identified; 11 RCTs fulfilled the inclusion criteria. VACs administered were diosmin, hidrosmin/rutosides, micronized purified flavonoid fraction (MPFF), and sulodexide. The studies included a highly variable proportion of patients with PTS (8.6%-100%). Some older studies omitted details of the methodology. Two studies suggested benefit of diosmin and MPFF as adjunctive treatment to rivaroxaban in the prevention of PTS and showed low or unclear risk of bias. Evaluation of RCTs for the treatment of post-thrombotic chronic venous insufficiency found low or unclear risk of bias in 81.6% to 85.7%. All studies suggested that VACs were beneficial for PTS treatment; they improved venous symptoms, decreased edema, and helped heal venous ulcers.

**Conclusions:**

This systematic review found that VACs had at least moderate quality of evidence in improving venous symptoms, decreasing edema, and accelerating venous ulcer healing. Two pilot RCTs of higher quality suggested the usefulness of diosmin and MPFF as adjunctive treatment to rivaroxaban therapy to reduce the incidence of PTS and improve deep vein recanalization. Because most RCTs were published over two decades ago, and several lacked the required precision in reporting, new high-quality, low-bias RCTs are needed to assess the role of specific VACs for both prevention and treatment of PTS.

Post-thrombotic syndrome (PTS) is a type of chronic venous insufficiency (CVI) that develops after a lower extremity deep vein thrombosis (DVT). PTS is highly prevalent; in one study (RIETE—Registro Informatizado Enfermedad Tromboembólica)^1^ at 1-year follow-up, 47.6% of patients with distal DVT (femoral/popliteal or tibial) developed PTS vs 60.5% of patients after iliofemoral or popliteal DVT. PTS is a debilitating condition caused by chronic inflammation and injury to the deep vein walls and valves. The proportion of PTS etiology in patients with venous leg ulcer (VLU) is significant. In the last Olmsted county venous ulcers epidemiological study,^2^ 36.6% of patients with VLU had PTS. One meta-analysis^3^ found that 41% of patients with VLU had PTS.

Clinical presentation of PTS may include varicose veins, edema, skin changes, and VLUs. PTS can cause significant pain and disability.^4^ It results in decreased health-related quality of life (QoL), increased risk of recurrent DVT (24% at 5 years), increased risk of developing a VLU, and high costs to the health care system for treatment and hospitalizations.^5^, ^6^, ^7^ The clinical manifestations are more advanced when extensive chronic obstruction and valvular incompetence (reflux) are present.^5^, ^6^, ^7^

Venoactive compounds (VACs) are derived from a variety of plants, trees, fruit, seeds, and flower extracts, and they can also be synthetic compounds. There are four main classes. The first is the benzopyrones, which are subdivided into the alpha benzopyrones with the active substance coumarin and the gamma benzopyrones or flavonoids with the active substances diosmin, micronized purified flavonoid fraction (MPFF), rutin, and rutosides. The second class is the saponins, which have escin and ruscus extract as active ingredients. The third class includes other plant extracts such as anthocyans, proanthocyanidins, and ginkgo biloba. The fourth class is synthetic compounds such as calcium dobesilate, benzarone, and naftazone.^8^, ^9^, ^10^ All VACs have various mechanisms of action in producing their clinical effects. The overarching mechanism is an increase in venous tone; other mechanisms involve their anti-inflammatory properties, inhibition of leukocyte adhesion and migration, improvement of lymphatic drainage, and enhancement of endothelial cells’ functionality, including their effect on blood flow rheology^8^, ^9^, ^10^ and protection of the endothelial glycocalyx.^11^

A different class of drugs is the glycosaminoglycans, which have anti-inflammatory, antithrombotic, and profibrinolytic pharmacologic properties, and also help restore the function of the glycocalyx.^12^, ^13^, ^14^ Sulodexide is made of repeating disaccharide units of unbranched polysaccharides that are highly sulfated and composed of 80% heparan sulfate (glucuronic acid and glucosamine) and 20% dermatan sulfate (iduronic acid and galactosamine).^12^^,^^15^

Pharmacologic treatment using VACs is an important component in the management algorithm of chronic venous disease (CVD). VACs have been helpful in relieving symptoms such as pain and leg heaviness, decreasing edema, and healing VLUs in conjunction with compression therapy.^9^^,^^10^^,^^16^

VACs have been widely used in Europe and in many countries outside the United States. In the United States, VACs are available as nutritional supplements. The information on the benefits of VACs in PTS prevention and treatment has been mixed with data on primary, nonthrombotic venous disease.^17^, ^18^, ^19^ The objective of our study was to perform a systematic review to determine the quality of evidence on the utility of VACs for both prevention and treatment of PTS.

## Methods

**Fig 1:**
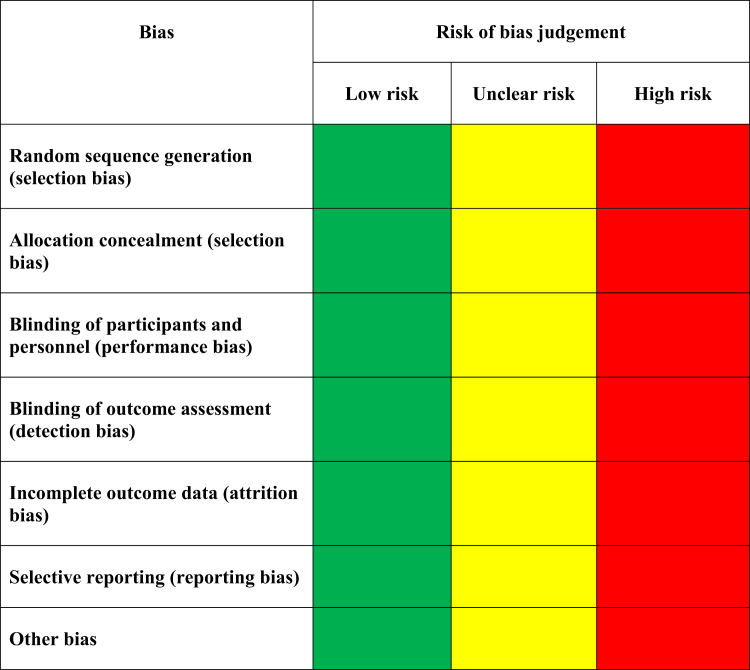
Cochrane reviews evaluation criteria for randomized controlled trials (RCTs).^21^

A systematic review was performed by searching articles for venoactive drugs or medications, DVT, and PTS using PubMed, MEDLINE, life science journals, and the Cochrane Library, published between January 1, 1980, and July 14, 2023. The following search terms were used from the Medical Subject Headings thesaurus: calcium dobesilate, daflon, diosmin, escin, flavonoids, horse chestnut, hydroxyethylrutoside, Pycnogenols, ruscus, sulodexide, troxerutin and postthrombotic syndrome, or deep vein thrombosis. Other search text words were phlebotonics or venoactive drugs/medications, micronized purified flavonoid fraction (MPFF), and red vine leaf extract. Only randomized controlled trials (RCTs) published in English language were included in this review that followed recommendations of the Preferred Reporting Items for Systematic Reviews and Meta-Analyses (PRISMA) guidelines^20^ and the revised Cochrane risk-of-bias tool for RCTs (see Fig 1).^21^ Four authors (MG, JS, AP, and JR) reviewed the manuscripts and selected the RCTs that were included in this review. They also judged the biases of the studies and attributed low, unclear, or high risk-of-bias assessment. The comparisons of evaluations were discussed until the agreement was reached. In case of disagreement of opinions, a fifth reviewer (PG) solved the discrepancy. The percentages of low, unclear, and high risk-of-bias assessments were calculated for all RCTs. A review of the selected articles was then performed with analyses of clinical presentation, PTS diagnostic methods, VAC treatment duration, type of drugs used, and dose, and evaluation of primary and secondary outcomes, including any side effects. Descriptive statistics were used.

## Results

**Fig 2:**
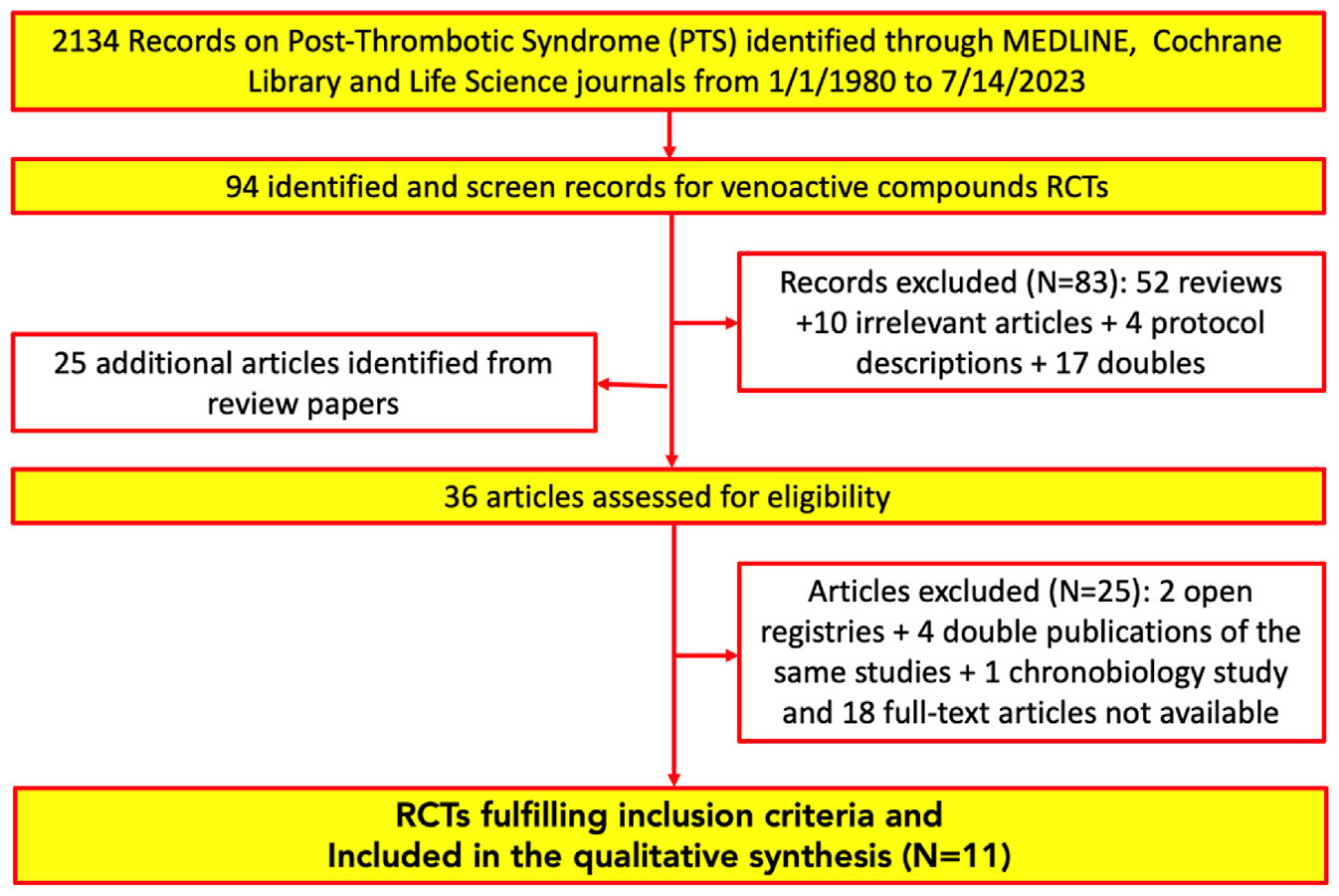
Preferred Reporting Items for Systematic Reviews and Meta-Analyses (PRISMA) flow diagram. *RCT*, Randomized controlled trial.

Eleven RCTs fulfilled the inclusion criteria (see Fig 2).

### Prevention of PTS

Two recent RCTs (from 2019 and from 2020)^22^^,^^23^ were identified with relatively low risk of bias, one for diosmin and one for MPFF (see Table I). Both studies were performed in the same medical center and included 90 and 60 patients, respectively, with first isolated femoropopliteal DVT. To prevent PTS, VACs were given as adjuvant therapy during and after acute DVT. Protocols had 6- and 12-month follow-up. The evaluation was done by a blinded assessor. Diosmin 600 mg^22^ and MPFF 1000 mg^23^ daily were added to standard treatment with rivaroxaban, and compression was used in each patient.

**Table I:** Randomized controlled trials (*RCTs*) administering venoactive compounds (*VACs*) for primary prevention of post-thrombotic syndrome (*PTS*) in patients with deep vein thrombosis (*DVT*)

First author/year	Methodology	Number of PTS patients (%)	PTS assessment	Outcomes	Complications and adverse events (AEs)	Dropout
Schastlivtsev 2020^22^	Single-center open-label RCT, 90 (45 × 2) patients with first femoropopliteal DVT. *Control group*: standard therapy rivaroxaban (15 mg × 2 for 3 weeks and 20 mg OD up to 6 months) + elastic compression stockings 12 months (23-32 mm Hg, 24 hours first week and during the day later). *Diosmin group*: diosmin 600 mg plus standard therapy. *Blinded assessor*. *Interim analysis* (50% of enrolled patients reaching 6 months).	PTS (Villalta >5) *At 6 months*: 6 (13.3%) vs 23 (51.1%) in the control group. Total 29/90 (32.2%) *At 12 months*: 4 (8.9%) vs 22 (48.9%) in the control group. Total 26/90 (28.9%)	Villalta score	• PTS assessment by the Villalta score at 12 months (primary outcome) • Reduction in the Marder score • Vein recanalization (clearance of thrombotic mass ≥80%) • VCSS and CIVIQ-20 scores • DVT recurrence • Adverse events	Clinically relevant nonmajor or minor bleeding: 3/45 (6.7%) vs 4/45 (8.9%) Other AEs: 2/45 (4.4%) vs 0/45 (0%)	0
Lobastov 2019^23^	Single-center open-label pilot 6-month RCT, 60 (30 × 2) patients with first isolated FP DVT. *Control group*: standard therapy rivaroxaban (15 mg × 2 for 3 weeks and 20 mg OD up to 6 months) + elastic compression stockings 6 months (23-32 mm Hg, 24 hours first week and during the day later). *MPFF group*: adjunctive MPFF 500 mg/twice a day (1 g). *Blinded assessor*. *DUS* every 2 months.	6/30 (20%) and 17/30 (57%). Total 23/60 (38.3%)	Villalta score	• PTS assessment by the Villalta score at 6 months • VCSS score • Reduction in the Marder score • Rate of recanalization for the FV and the PV • Bleeding complications and AE • DUS every 2 months	Clinically relevant nonmajor or minor bleeding: 2/30 (6.7%) vs 3/30 (10%) Other AEs: 3 (mild dyspeptic disorders with MPFF)/30 (10%) vs 0/30 (0%)	0
Total		*At 6 months*: VAC 12/75 = 16%, Control 40/75 = 53.3%, Total 52/150 = 34.7% *At 12 months*: VAC 4/45 = 8.9%, Control 22/45 = 48.9%, Total 26/90 = 28.9%				

*CIVIQ-20*, Chronic Venous Disease Quality of Life Questionnaire-20; *DUS*, duplex ultrasound; *FP*, femoro-popliteal; *FV*, femoral vein; *MPFF*, micronized purified flavonoid fraction; *OD*, once daily; *PV*, popliteal vein; *VCSS*, Venous Clinical Severity Score.

PTS, as defined by a Villalta score >5, was present at 12 months in 8.9% cases in the diosmin group vs 48.9% in the control group (relative risk: 0.14, 95% confidence interval: 0.04-0.43, *P* < .001). The diosmin group improved QoL, as assessed by Chronic Venous Disease Quality of Life Questionnaire-20 (22.1 ± 3.3 vs 28.8 ± 7.6, *P* < .01).

In the MPFF study, PTS developed at 6 months in 20% of the experimental group vs 57% in the control group (*P* = .007). VAC adjuvant treatment in both studies was associated with faster recanalization, higher rate of full recanalization, and lower Venous Clinical Severity Scores. No major bleeding was reported, and minor bleeding was equally distributed between the treated and the control groups. Mild dyspeptic disorders, which did not require treatment discontinuation, were found in 10% of patients in the MPFF group vs 0% for controls (*P* = .237). The two studies concluded that flavonoids added to conventional treatment improve clinical and ultrasound outcomes and decrease the risk of PTS after femoropopliteal DVT.

### Treatment of PTS

We identified nine RCTs that studied the effect of VACs for the treatment of PTS (Table II). Four studies included patients with various stages of CVD,^24^, ^25^, ^26^, ^27^ and five studies included only patients with VLUs, with CEAP (Clinical presentation, Etiology, Anatomy, Pathology) class C6.^28^^,^^29^^,^^31^, ^32^, ^33^ The total number of patients was 1091, of whom 36.5% had PTS.

**Table II:** Randomized controlled trials (*RCTs*) administering venoactive compounds (*VACs*) for the treatment of post-thrombotic syndrome (*PTS*)

First author/year	Methodology	Number of PTS patients (%)	PTS assessment	Outcomes	Complications and adverse events (AEs)	Dropout
Studies in patients with various stages of chronic venous disease						
Cospite 1989^24^	Double-blind RCT with MPFF vs diosmin. MPFF group 43 patients: functional venous insufficiency (FVI) 21+ varicose veins (VV) 12 + post-thrombotic syndrome (PTS) 10 (23.3%). Diosmin group 45 patients: FVI 18 + VV 20 + PTS 7 (15.6%).	10/43 (23.3%) in the MPFF group, 7/45 (15.6%) in the diosmin group Total 17/88 (19.3%)	NA	• Leg heaviness, pain, heat sensation, and skin redness (4-degree scale: 0 none to 3 severe). • Ankle and calf circumferences. • Safety: AE. • Clinical acceptability: patients’ satisfaction. • Plethysmography parameters: maximum increase of venous volume MVIV 40 and 60, total time for emptying tTE 40, distensibility dV/dP, dP/dV.	• 7 cases of epigastric pain (MPFF) vs 5 (diosmin)	2 (1 in each group)
Monreal 1994^25^	Prospective, open cross-over study comparing hidrosmina 200 mg/3 times a day (600 mg) to rutosides 300 mg 3 times a day (900 mg/d) in patients with PTS. Patients were treated for 6 months and then switched to the other drug for another 6 months. Symptoms were evaluated according a 10-point scale based on edema, pain, pigmentation, and ulcers. All patients wore compression hose. Group I (15 patients): hidrosmina-rutosides. Group II (14 patients): rutosides-hidrosmina. Follow-up visits at 3 and 6 months.	29/29 (100%)	Unilateral deep vein insufficiency of >12 months’ duration in patients with DVT in the affected leg diagnosed by venography. Minimum 20 mm baseline difference in ankle and calf circumference between the affected leg and other leg.	• Pain and tiredness. • Ankle and calf circumference. • Ulcer healing.	NA	0
Gilly 1994^26^	Double-blind, RCT vs placebo, 2 centers. Patients with symptomatic disturbances of the veno lymphatic system, including chronic venous insufficiency. 8 weeks’ treatment: 2 tablets of either MPFF 500 mg (n = 80) or placebo (n = 80).	12/80 in each group 24/160 (15%)	History of DVT before the CVI development	• Evolution of symptoms (functional discomfort, sensation of heaviness, nocturnal cramps, sensation of swelling, pain and sensation of heat or burning) over the 2-month observation (primary end point) • Change of calf and supramalleolar circumference (secondary end point)	9 (MPFF) and 12 (placebo) AEs: • Nausea (4/group) • Gastralgia (2/group) • Headaches (1 vs 3) • Insomnia (1/group) • Hypotension (1/group) • Menometrorrhagia (1 for placebo)	3:1 (nausea) vs 2 (nausea + hypotension)
Tsouderos 1989^27^	3 RCTs vs placebo. One randomized double-blind cross-over phase 2 trial using MPFF in 20 patients with PTS.	30/90 (33%)	NA	Plethysmography • Venous capacitance (Hmax mmm) • Total emptying venous time • Time to active emptying Improvement of functional and objective symptomatology • Functional discomfort • Sensation of heaviness • Sensation of weakness • Burning sensation • Edema: reduction of malleolar perimeter	NA	NA
Studies in patients with venous leg ulcers						
Glinski 1999^28^	Open multicenter RCT in patients with venous ulcer receiving standard wound care + compression alone or MPFF (2 tablets = 1000 mg BID) + wound care and compression for 24 weeks.	12 (6 in each group)/140 (8.6%)	NA	• Ulcer healing rate • Mean reduction in ulcer size • Photographs taken and wounds measured by planimetry • Safety (AEs) • Cost analysis	NA	4/71 (5.6%) in the MPFF group and 17/69 (24.6%) in the control group (*P* < .05)
Guilhou 1997^29^^,^^30^	Double-blind, multicenter (9 centers), randomized, parallel groups, controlled vs placebo trial, stratification according to ulcer size. Compliance to continue the study (not to be excluded) 80%-120%. MPFF group n = 53. Placebo group n = 48. Ulcer size ≤10 cm in 91 patients (MPFF 44 and placebo 47). Ulcer >10 cm in 14 patients (MPFF 9 and placebo 5).	63 (33 in the placebo group and 30 in the MPFF group)/105 (60%)	NA	• Rate of ulcer at 2 months • Time to healing • Sensation of heaviness improvement • Hospitalization duration • Safety (AEs)	MPFF group: • 2 venous thromboses in the MPFF group: 1 popliteal asymptomatic on day 6, and a superficial phlebitis after 43 days • Skin changes around the ulcer (1) • Asthenia (1) • Headaches (1) • Exacerbation of chronic colopathy (1) Placebo group: • Eczema (2) • Urticaria (1) • Pruritus of the scalp (1) • Local pain (1)	• 2 from MPFF group no data • 2 from MPFF (1 phlebitis, 1 noncompliance) • 4 from the placebo group (3 mild cutaneous events +1 personal reason)
Roztocil 2003^31^	Multicenter RCT in 150 included patients (68 in the control group and 82 in the MPFF group) with venous leg ulcer ≥2 and ≤10 cm of diameter, 6-month ttt with standard local care + compression ± MPFF.	50/82 (MPFF, 61%) and 54/68 (control, 79.4%) Total 104/150 (69.3%)	History of DVT	• Primary end point: rate of healed ulcers • Secondary end points: time to healing, ulcer size reduction, appearance of the skin, and venous symptoms	• The acceptability in the MPFF group evaluated by patients was excellent for 84.9% and good for 15.1%.	• 11 lost to follow-up in the control group • Premature withdrawal 5 for the control and 2 for the MPFF group due to unrelated complications
Coccheri 2002^32^	RCT SUAVIS study vs placebo (venous arm) to evaluate the efficacy of sulodexide in healing ulcers in 235 patients (121 vs 114) observed for 3 months. 31 Italian centers. Sulodexide administration by IM injection 60 mg for 20 days, then 50 mg BID for 70 days. Compression bandages and local wound care in all patients. Area of ulcer measured at 3 weeks, 2 months, and 3 months.	46/121 (38%) 29/114 (25.4%) Total 75/235 (31.9%)	Previous DVT history	• Primary outcome: healing rate at 2 months • Secondary end points: healing at 3 months, change in ulcer area • Safety (AEs)	• 40 AEs in total: 23 (19.1%) for sulodexide (treatment related: cutaneous rash, diarrhea, epigastric pain, headache) and 17 (15.4%) for placebo (treatment related: new leg ulcer, cutaneous rash, epigastric pain, abdominal pain)	• 2/121 (sulodexide [S]) plus 8/114 (placebo) before treatment • 31/120 (S) • (25.8%, 6 after ulcer healed) and 24/110 (placebo) • (21.8%, 5 after ulcer healed)
Scondotto 1999^33^	RCT to evaluate efficacy of sulodexide vs control in healing ulcers at 2 months. Group wound care only (42 patients) vs wound care plus sulodexide IM 600 units × 30 days, followed by oral dose of 500 mg for another 30 days (52 patients).	24/52 (46.2%) and 20/42 (47.6%) 44/94 (46.8%)	Previous DVT history Diagnosis confirmed by echo Doppler	• Healing rate at 2 months • Healing time • Correlation with age, sex, etiology, and the severity and duration of the ulcer	NA	NA
Total		398/1091 (36.5%)				

*BID,* Twice a day; *CVI,* chronic venous insufficiency; *IM,* intramuscular; *MPFF,* micronized purified flavonoid fraction; *NA,* not applicable; *OD,* once daily; *tTE,* total time for emptying; *ttt,* treatment.

PTS was defined in 5 of 9 studies, and it was based on history of a previous DVT.^6^^,^^7^^,^^11^, ^12^, ^13^ In one study, patients with PTS were characterized by deep venous insufficiency of >12-month duration and increased leg circumference after DVT, which was confirmed with venography.^25^ In another study, PTS changes were confirmed by Duplex scanning.^33^

One pilot open cross-over study by Monreal et al^25^ focused on patients with PTS alone and compared the efficacy of 6-month therapy with hidrosmin, O-(β-hydroxyethyl) diosmin, and rutosides. Patients who received either of the compounds demonstrated a decrease in ankle and calf circumference, and improvement in ulcer healing and PTS symptoms. Discontinuation of the VAC treatment resulted in the aggravation of symptoms in 10 of 29 patients and recurrence of ulcers in 2 patients.

In a double-blind RCT, Cospite and Dominici^24^ found that 19.3% of the patients with CVI had PTS. The efficacy of MPFF was compared with that of nonmicronized diosmin. Symptomatic improvement, assessed by a 4-degree scale at 60 days, was significantly better in the MPFF group for leg heaviness, pain, heat sensation, and skin redness (*P* = .002-.04). Also, in the MPFF group, a significantly better decrease in ankle and calf circumferences (*P* < .001) was observed. Safety was similar in both groups, with epigastric pain that spontaneously resolved reported in 11.4% of cases for diosmin and 15.9% for MPFF (non-significant). Patients’ satisfaction was higher in the MPFF group (95% vs 80%, *P* < .01). Plethysmographic evaluation at the end of the study revealed, for both MPFF and diosmin, a significant improvement of maximum increase in venous volume (40 and 60), total time for emptying (40), distensibility (dV/dP), and bulk modulus or elasticity (dP/dV). The authors did not provide a separate analysis for patients with PTS.

Another double-blind placebo-controlled RCT by Gilly et al^26^ included 15% symptomatic patients with PTS, treated for 2 months. The primary end point was the evolution of symptoms. Compared with placebo, MPFF demonstrated improvement of symptoms including functional discomfort (mean scores: 0.5 vs 1.2 at 8 weeks, *P* < .001), sensation of heaviness (mean scores: 0.7 vs 1.3 at 8 weeks, *P* < .001), nocturnal cramps (mean scores: 0.3 vs 0.7 at 8 weeks, *P* = .002), sensation of swelling (mean scores: 0.5 vs 1.3 at 8 weeks, *P* < .001), pain (mean scores: 0.6 vs 0.9 at 8 weeks, *P* = .027), and sensation of heat/burning (mean scores: 0.3 vs 0.7 at 8 weeks, *P* = .006). The secondary end points (calf and supramalleolar circumferences reflecting edema) were significantly more decreased in the MPFF group (4.6 vs 1 mm at 4 weeks, *P* < .001; 7.1 vs 1.2 mm at 8 weeks, *P* < .001). The side effects (nausea, gastralgia, headaches, insomnia, hypotension, and menometrorrhagia) were reported in 9 of 76 (11.8%) patients from the MPFF group and 12 of 74 (16.2%) from the placebo group. No changes in laboratory parameters were observed. The conclusions stated that MPFF had early and prolonged efficacy in the treatment of patients with CVD. The authors did not provide separate analysis for patients with PTS.

Tsouderos^27^ reported the combined results of three double-blind RCTs of MPFF vs placebo. One cross-over study was conducted in patients with PTS, another in three groups of patients with varicose veins, venous insufficiency during pregnancy, and PTS, and a third RCT in patients with functional CVI. Altogether 33% of the patients had PTS. Using venous occlusion plethysmography, the author demonstrated that in all groups, MPFF significantly decreased mean venous capacitance and total emptying venous time (*P* < .001) and time to active emptying (*P* < .001) in 2 hours after single-dose administration. Venous symptoms (functional discomfort; sensation of heaviness, weakness, burning, and swelling) improved by 45% vs placebo after 1 month and by 73% to 75% after 2 months of treatment. A significant reduction of malleolar perimeter was observed in the MPFF group (*P* < .001).

Four of five RCTs performed in patients with venous ulcer included variable percentages of PTS, from 8.6% to 60%.^28^^,^^30^, ^31^, ^32^, ^33^ PTS was not specified as the etiology of venous ulcer in one study.^32^ Three RCTs included treatment with MPFF,^28^, ^29^, ^30^, ^31^ and two with sulodexide.^32^^,^^33^ In all cases, venoactive therapy was adjunctive to standard leg ulcer treatment (local wound care and compression therapy) and was compared either with placebo,^29^^,^^32^ or with a control group with standard protocol alone.^28^^,^^31^^,^^33^ The duration of therapy varied from 2 months^29^^,^^33^ to 6 months.^28^^,^^31^ All studies reported significantly better healing rates, and three reported significantly shorter time to healing.^28^^,^^30^^,^^33^ The post-thrombotic origin was assumed in patients with DVT based on the medical history,^31^, ^32^, ^33^ whenever the diagnostic criteria were provided, and in one study, the confirmation of the PTS changes by Duplex scanning was required.^33^ Treatment was equally effective for both primary and secondary VLUs.

All studies concluded that VACs were beneficial to improve venous symptoms, edema, and healing of venous ulcers.

### Assessment of RCT quality

**Fig 3:**
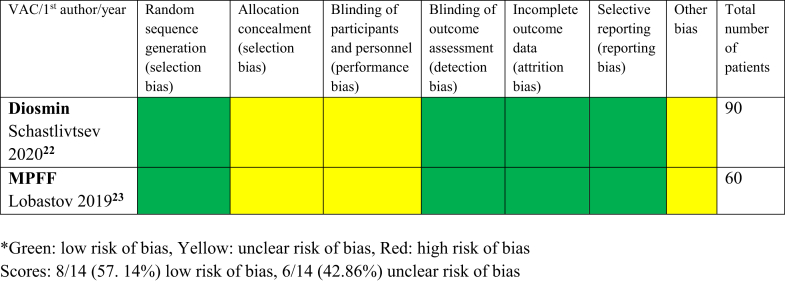
Quality of randomized controlled trials (RCTs) in post-thrombotic syndrome (PTS) prevention assessed using Cochrane review criteria. Scores: 8/14 (57.14%) low risk of bias and 6/14 (42.86%) unclear risk of bias. Green: low risk of bias, yellow: unclear risk of bias, and red: high risk of bias. *MPFF*, Micronized purified flavonoid fraction; *VAC*, venoactive compound.

Low or unclear risk of bias were predominant in two studies, with diosmin and MPFF administered for prevention of PTS^22^^,^^23^ (Fig 3). However, in these studies, allocation concealment and blinding of participants and personnel were not specified, and the manufacturer of the drugs funded the articles’ processing and publication fee (other bias). The strength of the two RCTs lies in the independence in the study performance, clear description of the random sequence generation, blinded outcome assessment, and complete outcome data and reporting.

**Fig 4:**
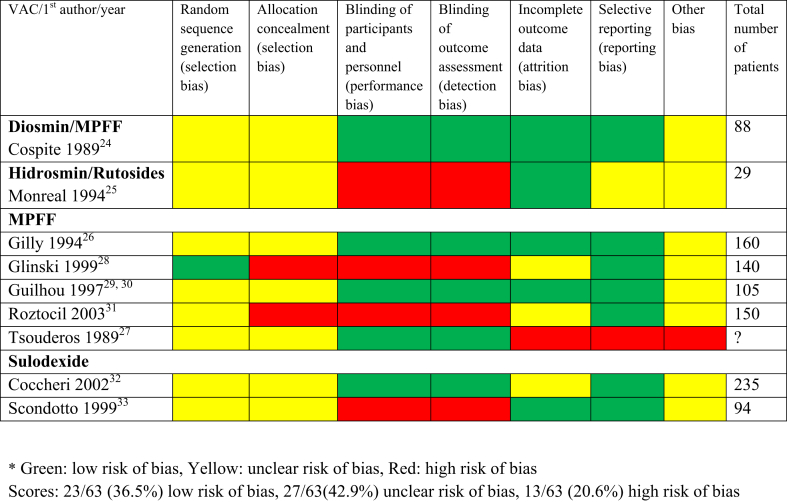
Quality of randomized controlled trials (RCTs) in post-thrombotic syndrome (PTS) treatment assessed using Cochrane review criteria. Scores: 23/63 (36.5%) low risk of bias, 27/63 (42.9%) unclear risk of bias, and 13/63 (20.6%) high risk of bias. Green: low risk of bias, yellow: unclear risk of bias, and red: high risk of bias. *MPFF*, Micronized purified flavonoid fraction; *VAC*, venoactive compound.

The evaluation of RCTs for the treatment of PTS (Fig 4) found low or unclear risk of bias in 71.4% to 100% of the criteria when diosmin, hidrosmin/rutosides, MPFF, and sulodexide were used. For these studies, randomization generation was described only in one^28^ of nine. We assessed risk of bias for allocation concealment as high in two open studies;^28^^,^^31^ the others did not provide clear situation either. The risks related to blinding of participants/personnel and blinding of outcome assessment were low in five^24^^,^^26^^,^^27^^,^^29^^,^^30^^,^^32^ of nine RCTs. Obviously, these older studies often failed to specify the methodology details as required by the Cochrane criteria. Unclear risks of incomplete outcome data^28^^,^^31^^,^^32^ or selective reporting^25^ were found in four studies, and high risk was found in one study.^27^ Other possible risks of biases included a small pilot study, no sample size calculations, and possible influence by the manufacturer of the drug.

Considering several risks of biases, the level of evidence of these RCTs should be downgraded to the moderate B level.

## Discussion

Current standard of care of patients with CVI due to PTS is compression treatment,^34^ in addition to venous interventions that decrease ambulatory venous pressure and improve venous return. However, patients frequently complain of discomfort and difficulties in donning compression garments. Kankam et al^35^ found that less than 50% of the patients with CVD are compliant with wearing compression. In many studies (19 of 58), compliance was not even evaluated.^35^ Skin irritation is the most frequent complication limiting the compliance and efficiency of compression therapy. Adding VACs to compression therapy likely allows wearing less compressive garments and improves the overall outcome.

The VEIN STEP, a prospective observational study,^36^ including 6084 patients with CVD, determined symptomatic improvement after 2 weeks in 89% of patients treated by conservative therapy, which included VACs, compression, and topical treatment of VLUs. Moreover, an analysis of patients with VLU^37^ reported 45% improvement in cost-effectiveness ratio when adjuvant MPFF treatment was combined with compression and local wound care. The superiority of the adjuvant VAC treatment with compression over compression alone was also demonstrated in patients with CVI with oxerutins.^38^ Comparison of compression stockings and another VAC, oral horse-chestnut seed extract therapy,^39^ concluded that both treatments provided similar antiedema protection.

The effect of sulodexide treatment in the management of DVT and PTS was confirmed in registries,^40^^,^^41^ one of them introducing a new compound, Pycnogenol, an extract from the bark of a European pine tree.^41^ However, no Pycnogenol RCTs were performed for PTS prevention or treatment. The only randomized trial with Pycnogenol in CVI, compared with MPFF,^42^ excluded patients with a history of thrombosis or the presence of recent thrombosis and was not conducted in a double-blind fashion. The authors concluded that Pycnogenol treatment was more effective than MPFF.

Connections between the arterial and venous system have been evident,^43^ demonstrating common pathophysiological processes affecting the endothelium and related risk factors in both arterial and venous diseases. Pentoxifylline, a xanthine derivative used in the management of peripheral vascular disease, is indicated in VLUs with level A recommendation based on high-quality RCTs and a Cochrane systematic review. Sulodexide and diosmin were found to have the protective glycocalyx restorative and anti-inflammatory effects on injured arterial vessels.^11^^,^^44^

A new RCT investigating the effect of MPFF in PTS, the MUFFIN-PTS trial,^45^ was recently launched by the Canadian Institute of Health Research. This RCT will include 86 patients with PTS randomized to oral MPFF (1000 mg daily) or placebo for 9 months in addition to the standard PTS therapy including elastic compression stockings, exercise training, lifestyle measures, and radiological interventions in severe cases. Primary outcome measure is improvement in Villalta score (proportion of patients with a decrease in score of at least 30%) for the affected leg.

Our systematic review has several limitations. This is a narrative review with descriptive statistics. A meta-analysis was not performed because of the small number of studies and a significant heterogeneity using multiple VACs for therapy. Many studies were published over two decades ago, with limitations in design and reporting. There was also a distinct difference in the quality of reporting studies in favor of the more recent trials^22^^,^^23^ vs those published from 1989 to 2003^24^, ^25^, ^26^, ^27^, ^28^, ^29^, ^30^, ^31^, ^32^, ^33^ (Figs 3 and 4).

Given this limited evidence, we should make a strong recommendation for future large, high-quality RCTs with proper randomization and blinding, standardized PTS diagnostic criteria and outcome measures (including QoL and patient-reported outcomes, and biomarkers’ studies), and long-term follow-up beyond 12 months. Ideally, these studies would include comparisons between different VACs and cost-effectiveness analyses. Also, it would be interesting to study specific patient subgroups such as iliofemoral vs femoropopliteal DVT, primary vs recurrent DVT, and different severity levels of PTS. Other elements to determine would be optimal timing and duration of VAC therapy and potential synergy between different therapies.

## Conclusions

This systematic review found that some VACs (diosmin, hidrosmin, MPFF, and sulodexide) had at least moderate level of evidence and in some RCTs high level of evidence in improving venous symptoms, decreasing edema, and accelerating venous ulcer healing in PTS. Diosmin and MPFF as adjunctive treatment to rivaroxaban therapy reduced the incidence of PTS after femoropopliteal DVT and increased deep vein recanalization. Most studies, however, were conducted many years ago; they had significant heterogeneity and some lacked reporting precisions. New, high-quality, low-bias RCTs are needed to assess the role of specific VACs for both prevention and treatment of PTS.

## Author contributions

Conception and design: MG, JS, AP, PG, JR

Analysis and interpretation: MG, JS, AP, PG, JR

Data collection: MG, JS, AP, JR

Writing the article: MG, JS, AP, PG, JR

Critical revision of the article: MG, JS, AP, PG, JR

Final approval of the article: MG, JS, AP, PG, JR

Statistical analysis: Not applicable

Obtained funding: Not applicable

Overall responsibility: MG

MG, JS, AP, and JR contributed equally to this article, and PG contributed in the concept, final review of the results, and preparation of the manuscript.

## Funding

None.

## Disclosures

MG is a scientific advisor to VitasupportMD company. The remaining authors report no conflicts.
